# RNA-mediated transgenerational inheritance in ciliates and plants

**DOI:** 10.1007/s00412-017-0655-4

**Published:** 2017-12-11

**Authors:** Zachary T. Neeb, Mariusz Nowacki

**Affiliations:** Institute of Cell Biology, University of Bern, Baltzerstrasse 4, 3012 Bern, Switzerland

**Keywords:** Transgenerational inheritance, Non-Mendelian inheritance, Small RNA, Epigenetics, Ciliates, Plants

## Abstract

In the age of next-generation sequencing (NGS) and with the availability of whole sequenced genomes and epigenomes, some attention has shifted from purely sequence-based studies to those of heritable epigenetic modifications. Transgenerational inheritance can be defined as heritable changes to the state of DNA that may be passed on to subsequent generations without alterations to the underlying DNA sequence. Although this phenomenon has been extensively studied in many systems, studies of transgenerational inheritance in mammals and other higher-level eukaryotes may be complicated by the fact that many epigenetic marks are reprogrammed during sexual reproduction. This, by definition, may obscure our interpretation of what is in fact truly transgenerational. Therefore, in this mini review, we discuss what is currently known in the field about transgenerational epigenetic inheritance in ciliates and plants, with a particular emphasis on RNA-mediated processes and changes in chromatin states.

## Introduction

In recent years, there has been much focus on the subject of epigenetic inheritance and how heritable changes in chromatin states may be transmitted to subsequent generations. This includes changes in gene activity and gene expression levels, without altering the underlying DNA sequence, that may be passed on to generations to come. Transgenerational inheritance may involve DNA methylation or other chromatin-based mechanisms, but can also involve RNA-mediated DNA methylation and RNA-mediated DNA excision/elimination in some of the more extreme examples. Indeed, small non-coding RNAs have been implicated in many of these processes and likely mediate transgenerational inheritance across eukaryotic species, since they can induce changes in chromatin dynamics and guide histone modifications. While these phenomena have been described in mammals and extensive work has been performed to elucidate mechanisms, this review will focus primarily on RNA-mediated transgenerational inheritance in ciliated protozoans and plants.

## Ciliates

Ciliates are large, unicellular protists that can be found ubiquitously across the globe in both marine and freshwater environments. Ciliates exhibit a special case of germline-soma specialization called nuclear dimorphism and are thus unique systems to study RNA-mediated transgenerational inheritance. Ciliates contain two completely separate caches of genetic information: the micronucleus (MIC), considered the germline nucleus, is transcriptionally silent and is used to propagate genetic information from one generation to the next and the macronucleus (MAC), considered the somatic nucleus, is used for vegetative growth of the cells (Prescott 1994). The micronuclear genome resembles that of a canonical eukaryotic genome, with many genes organized along long chromosomes. The micronuclear genome contains a large amount of “junk” DNA including transposable elements (TEs) and repetitive elements such as minisatellites, while micronuclear genes themselves are often interrupted by multiple short transposon-derived stretches of non-coding DNA called internally eliminated sequences (IESs) (Arnaiz et al. 2012; Chen et al. 2014; Guerin et al. 2017; Hamilton et al. 2016). The macronuclear genome, on the other hand, is devoid of all of this “junk” DNA and all the transcription necessary for vegetative growth occurs here (Aury et al. 2006; Duret et al. 2008; Eisen et al. 2006; Fang et al. 2012; Swart et al. 2013). During the ciliate sexual life cycle, the parental macronucleus provides genetic information in the form of transported sRNAs for the formation of a new macronucleus, which is derived from a newly formed, micronuclear precursor. During this micronucleus to macronucleus transition, the micronuclear genome is modified drastically through various processing events, including the polytenization of chromosomes and removal of repetitive DNA sequences, and IESs must be precisely removed to create functional macronuclear genes. It has been shown that small RNAs (sRNAs) are involved in the epigenetic transmission of information from parental nuclei to the developing macronucleus, leading to large-scale genomic rearrangements, altered chromatin states and ultimately the complete removal of specific DNA sequences.

In the stichotrich *Oxytricha trifallax*, approximately 20% of micronuclear genes exist in a non-linear, “scrambled” order that must be connected upon IES removal during macronuclear development (Chen et al. 2014). This means that in addition to targeting specific IES regions for elimination from the genome, the cells must also sort and reorder the remaining macronuclear destined sequences (MDSs) into functional genes. During *Oxytricha* conjugation, the parental macronucleus is broken down and degraded, while a new macronucleus, called the anlage, develops from one of the parental micronuclei. At this developmental stage, the anlage undergoes endoreplication, eliminates over 90% of its germline genome, breaks apart and fragments its chromosomes, and then ligates the thousands of remaining MDSs back together into functional genic reading frames (Adl and Berger 2000). A conjugation-specific class of 27 nt small RNAs called 27macRNAs has been identified and is highly upregulated during this process (peaking 24 h post-mixing of complementary mating types) (Fang et al. 2012; Zahler et al. 2012). These 27macRNAs are derived from the parental macronucleus, possess a strong 5′ U bias, and play a vital role during macronuclear development. The 27macRNAs associate with a PIWI protein called Otiwi1 and have been implicated in specifying which regions of the genome are protected from the DNA elimination occurring during this time (Fang et al. 2012). Microinjection of synthetic sRNAs corresponding to IES regions that are usually eliminated led to their retention in subsequent generations. Although little is known about the biogenesis of these RNAs or the exact mechanism by which DNA is protected, it has been suggested that this may occur through methylation of cytosine residues within IES regions (Bracht et al. 2012). In addition to the 27macRNAs necessary for DNA retention throughout macronuclear development, long maternal guide RNA templates transcribed from macronuclear nanochromosomes have also been shown to mediate genomic rearrangements (Nowacki et al. 2008). Long RNA transcripts (both sense and antisense), corresponding to entire macronuclear DNA molecules, can be detected for a brief period during conjugation and it is hypothesized that these act as templates for the correct unscrambling of MDSs. Microinjection of synthetic double-stranded nanochromosomes (DNA or RNA versions) with alternatively arranged MDSs led to defects in the proper reordering of MDSs in subsequent generations, indicating epigenetic inheritance through these RNAs (Nowacki et al. 2008). In a recent study, RNA-cached copies of over half of *Oxytricha* nanochromosomes have been identified during macronuclear development, supporting the model in which maternal guide RNA templates are transmitted to the progeny (Lindblad et al. 2017). Although it has been suggested that the long guide RNAs may act as precursors for the biogenesis of 27macRNAs, the relationship between these two classes of RNAs remains unknown. Interestingly, a striking number of the genes upregulated during *Oxytricha* macronuclear development encode well-conserved proteins with links to germline function and development in higher-level eukaryotes (Neeb et al. 2017).

**Fig. 1:**
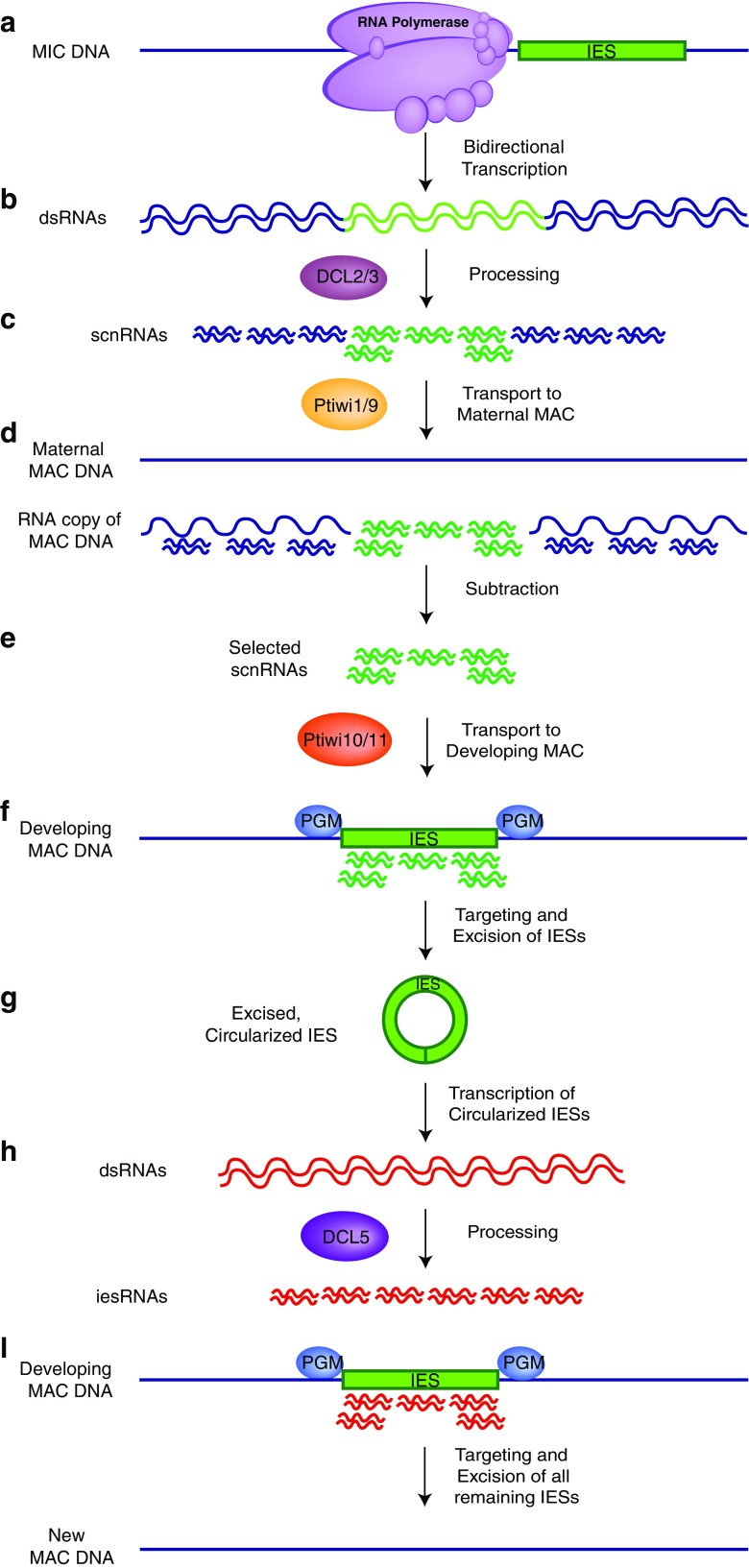
Scanning model for DNA elimination in *Paramecium tetraurelia*. (a) The micronuclear genome is transcribed bidirectionally by an unknown RNA polymerase to produce long double-stranded RNAs. (b) These long dsRNA precursors are processed by the Dicer-like enzymes DCL2/3 to produce 25 nt long scnRNAs. (c) scnRNAs, in association with the PIWI proteins Ptiwi1/9, are transported to the maternal macronucleus (MAC). (d) scnRNAs “scan” the macronuclear genome via interaction with RNA transcripts of somatic DNA. scnRNAs pairing to homologous macronuclear destined sequences (MDSs) are filtered out and degraded, leaving only those corresponding to germline internal eliminated sequences (IESs). (e) Selected scnRNAs, in association with PIWI proteins Ptiwi10/11, are transported to the developing MAC. (f) These scnRNAs target the excision of IESs by the excisase PiggyMac (PGM). (g) Excised IESs circularize, or concatemerize and circularize, and are transcribed into long dsRNAs. (h) These long dsRNA precursors are processed by the Dicer-like enzyme DCL5 to produce 22–31 nt long iesRNAs. (i) iesRNAs ensure the precise and efficient excision of all remaining IESs from the developing macronuclear genome. Development of the new MAC is completed, with the newly formed macronuclear genome matching that of the maternal macronucleus

In the more well studied ciliates *Paramecium* and *Tetrahymena*, it has also been shown that epigenetic information from the parental macronucleus guides the elimination and subsequent retention of specific DNA sequences during macronuclear development (Fig. 1) (Aronica et al. 2008; Lepere et al. 2008; Mochizuki et al. 2002). During the early stages of the sexual life cycle of these ciliates, the entire micronuclear genome is transcribed bidirectionally to produce long double-stranded RNAs (Chalker and Yao 2001; Mochizuki and Gorovsky 2004b). These double-stranded RNA precursors are then processed by Dicer-like enzymes, DCL2/3 in *Paramecium* and Dcl1p in *Tetrahymena*, to produce a class of small RNAs called scan RNAs (scnRNAs) (25 nt and 26–31 nt, respectively) (Chalker et al. 2005; Malone et al. 2005; Mochizuki and Gorovsky 2004a, 2005; Sandoval et al. 2014). These scnRNAs are then transported to the maternal macronucleus where they “scan” the macronuclear genome. Although the mechanism of this genome “scanning” is unknown, it is thought to involve interaction between transported scnRNAs and maternal RNA transcripts present in the developing macronucleus (Aronica et al. 2008; Lepere et al. 2008; Mochizuki et al. 2002). scnRNAs with homologous macronuclear sequence are degraded by an unknown mechanism, leaving only those corresponding to IESs remaining. According to the current model, the scnRNAs that survive this filtering step are transported to the developing macronucleus, where in association with PIWI proteins (Ptiwi1/9 in *Paramecium* and Twi1p in *Tetrahymena*) they are hypothesized to mark IESs for excision and elimination (Bouhouche et al. 2011; Duharcourt et al. 1995; Mochizuki et al. 2002; Mochizuki and Gorovsky 2004a; Sandoval et al. 2014). In both *Paramecium* and *Tetrahymena*, this elimination relies on repressive heterochromatin marks, namely histone H3 lysine 9 and lysine 27 methylation (Kataoka and Mochizuki 2011; Lhuillier-Akakpo et al. 2014; Liu et al. 2007; Mochizuki and Gorovsky 2004a; Taverna et al. 2002; Yao and Chao 2005). Excision of IES regions is facilitated by a domesticated *piggyBac* transposase (called PiggyMac in *Paramecium*), an endonuclease that creates DNA double-stranded breaks at MDS/IES junctions, and flanking MDSs are then joined by the protein DNA ligase IV (Baudry et al. 2009; Cheng et al. 2010; Dubois et al. 2012; Kapusta et al. 2011). In *Paramecium*, excised IESs circularize to become templates for the transcription of a second class of RNAs called iesRNAs (Allen et al. 2017; Betermier et al. 2000; Kapusta et al. 2011). These 22–31 nt small RNAs, complementary to the sequence of excised IESs, are produced by the Dicer-like enzyme DCL5 and act as a quality control mechanism to ensure the precise and accurate removal of all remaining IESs from developing macronuclear chromosomes (Sandoval et al. 2014). A second class of scnRNAs called late-scnRNAs, expressed later in macronuclear development, has also been reported in *Tetrahymena*. These late-scnRNAs are transcribed from IESs prior to their excision and can recognize not only the IESs from which they are transcribed, but also other IESs in *trans* (Noto et al. 2015). The mechanisms by which these lately expressed sRNAs recognize IESs within the developing macronuclear genomes remain to be elucidated; however, *Paramecium* iesRNAs have been recently shown to bind the previously unclassified PIWI proteins Ptiwi10 and Ptiwi11 (Furrer et al. 2017). It is worth noting that while the *Paramecium* micronuclear genome contains close to 45,000 IESs, the majority of which interrupt protein-coding regions, *Tetrahymena*, have significantly less (~ 8000) with very few contained within protein-coding genes (Arnaiz et al. 2012; Fass et al. 2011).

## Plants

**Fig. 2:**
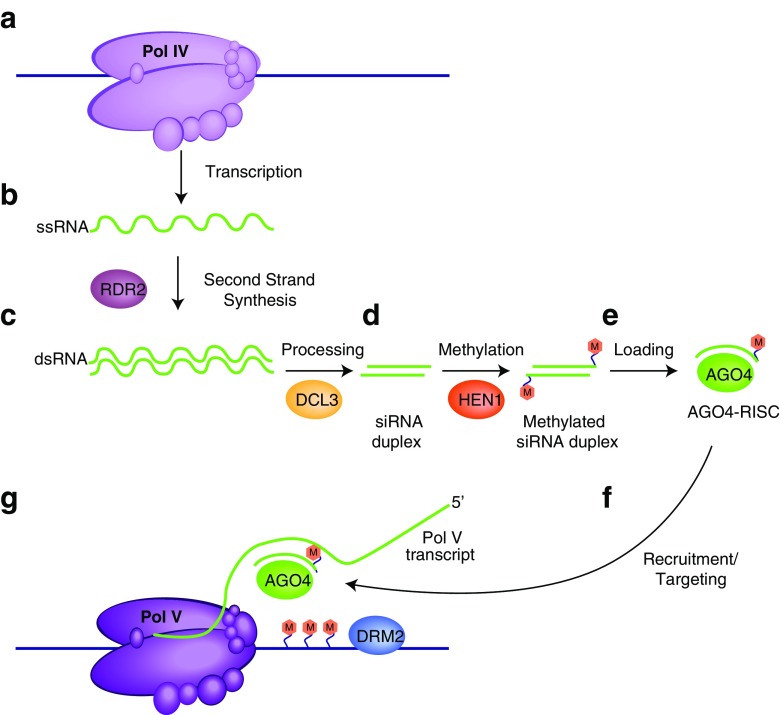
RNA-directed DNA methylation (RdDM) in *Arabidopsis thaliana*. (a) RNA polymerase IV produces single-stranded RNA transcripts that initiate the process of RdDM. (b) These single-stranded RNAs are used as templates for the transcription of a second strand by the RNA-dependent RNA polymerase RDR2. (c) These double-stranded RNA duplexes are cleaved by the Dicer-like enzyme DCL3 to produce 24 nt siRNAs. (d) 24 nt siRNA duplexes are modified by the RNA methylase HEN1, which adds a stabilizing 3′-O-methyl group. (e) A single 24 nt strand of RNA is loaded onto the Argonaute family protein AGO4 to form an active RNA-induced silencing complex (RISC). (f) This sRNA bound RISC complex is then recruited to growing transcripts produced by RNA polymerase V, where direct interaction between AGO4-bound sRNAs and nascent transcripts is thought to occur. (g) De novo cytosine methylation of the corresponding DNA sequence is mediated by the methyltransferase DRM2. This ultimately leads to the removal of active histone marks and the establishment of repressive ones, leading to silencing of specific genomic regions

Epigenetic inheritance has been most well studied in plants and generally involves heritable changes in DNA methylation states. Although extensive work has been performed to describe transgenerational silencing of transgenes in plants such as toadflax, tomato, and maize, here we will focus primarily on RNA-directed DNA methylation (RdDM) in the plant *Arabidopsis thaliana*. Interestingly, compared to mammals, plants are particularly prone to epigenetic inheritance even though both types of genomes tend to be saturated with TEs and other repetitive DNA sequences that must be silenced (Quadrana and Colot 2016). *Arabidopsis* and other flowering plants exhibit the best characterized example of duplication and functional specification of subunits of the RNA polymerase II complex. In addition to the canonical RNA polymerase II machinery, nearly universally composed of 12 core subunits in eukaryotes, *Arabidopsis* possesses two additional nuclear multi-subunit RNA polymerases, named RNA polymerase IV (Pol IV) and RNA polymerase IV (Pol V) (reviewed in (Haag and Pikaard 2011) (Kornberg 2007). These plant-specific RNA polymerases have non-redundant roles in RNA-mediated gene silencing pathways, specifically in RNA-directed DNA methylation (RdDM) that targets TEs and other repetitive sequences (Matzke and Mosher 2014; Tucker et al. 2010). Pol IV is responsible for transcribing short primary RNA transcripts, which are copied into dsRNAs by an RNA-dependent RNA polymerase, RDR2 (Blevins et al. 2015; Kasschau et al. 2007; Xie et al. 2004; Zhai et al. 2015; Zhang et al. 2007). After these double-stranded substrates are cleaved by the Dicer-like enzyme DCL3 to produce 24 nt siRNAs, they are stabilized by a 3′ end modification (2′-O-CH_3_ group) added by the methylase HEN1 (Li et al. 2005; Qi et al. 2005; Xie et al. 2004; Yu et al. 2005). These stabile siRNAs then associate with the Argonaute family protein AGO4 (or sometimes AGO6 and AGO9) to form an RNA-induced silencing complex (RISC) (Blevins et al. 2015; Havecker et al. 2010; Qi et al. 2006; Zhai et al. 2015). Pol V produces nascent long non-coding RNA (lncRNA) transcripts from specified regions of the genome which are hypothesized to base pair with the AGO4-associated siRNAs and this results in de novo cytosine methylation of the corresponding DNA template by the DNA methyltransferase DRM2 (Haag et al. 2009; Wierzbicki et al. 2008; Wierzbicki et al. 2009; Zhong et al. 2014). This often leads to gene silencing through repressive histone modifications (Kanno et al. 2010; Law and Jacobsen 2010) (Fig. 2). Proteomic analyses have revealed that *Arabidopsis* Pol IV and Pol V have a 12-subunit composition like Pol II. In fact, half of the subunits of Pols II, IV, and V are encoded by the same genes. The remaining Pol IV- or Pol V-specific subunit genes arose through duplication and subfunctionalization of ancestral Pol II subunit genes (Haag and Pikaard 2011). Unique paralogs of the largest subunit of Pol II (NRPB1) make up the catalytic core of the polymerases and are unique to either the Pol IV or Pol V complex, being referred to as NRPD1 and NRPE1, respectively (Herr et al. 2005; Kanno et al. 2005; Onodera et al. 2005; Pontier et al. 2005). While the NRPB1 C-terminal domain (CTD) contains heptapeptide repeats, the CTDs of both NRPD1 and NRPE1 lack this signature, likely facilitating their alternative functions. The NRPE1 CTD is extended by approximately 300 amino acids and is shown to associate with AGO4 through WG/GW repeats, called the Argonaute “hook,” to direct DNA methylation (Li et al. 2006).

To be considered truly transgenerational, these DNA methylation landscapes must be heritable and maintained in subsequent generations after their initial establishment. Maintenance of DNA methylation patterns through DNA replication requires the cooperation of several protein factors (Law and Jacobsen 2010). In *Arabidopsis*, DNA methylation is well maintained across TEs and genes and relies primarily on the de novo DNA methyltransferase (DMTase) MET1 for maintenance of CG methylation (Vongs et al. 1993). For maintenance of CHG methylation, an additional chromomethylase called CMT3 is necessary and specifically binds histone H3 lysine 9 dimethylation, while asymmetric CHH methylation maintenance relies on DRM2 and CMT2, only requiring CMT3 at specific loci (Cao et al. 2003; Du et al. 2015; Johnson et al. 2007; Lindroth et al. 2001; Lindroth et al. 2004; Stroud et al. 2014; Stroud et al. 2013). Evidence suggests that methylation patterns across TEs and repetitive sequence elements are transmitted from parent to offspring upon fertilization, although CHH methylation must be reestablished as the embryo develops (Hsieh et al. 2009; Jullien et al. 2012). CHH methylation is guided by maternally inherited 24 nt siRNAs that are present upon fertilization (Calarco et al. 2012; Lu et al. 2012; Mosher et al. 2009). Plants tend to undergo significantly less germline reprogramming of DNA methylation patterns than mammals, displaying an excellent example of transgenerational epigenetic inheritance (Heard and Martienssen 2014).

## Perspectives

Both plants and ciliates exhibit a case of RNA-mediated epigenetic inheritance, utilizing classes of small RNAs, but the mechanisms by which they perform such a feat are quite different. While RNA polymerase IV and RNA polymerase V are involved in the transcription of sRNA precursors and the nascent transcript targets in plants, this process is much less clear in ciliates. Plants use an RNA-dependent RNA polymerase (RDRP) to transcribe the second strand of RNA before Dicer-like cleavage, but it is hypothesized that in ciliates, transcription occurs bidirectionally to form dsRNA substrates, although the polymerase responsible has yet to be identified. Interestingly, however, additional RNA polymerase II subunits have also been identified in ciliates that may play similar roles to plant Pols IV and V. For example, *Oxytricha* has additional largest and second largest Pol II subunit paralogs (RPB1b and RPB2b) that are highly upregulated during macronuclear development and likely play roles in the transcription of either small RNA precursors or the guide RNAs described in the process of gene unscrambling (Khurana et al. 2014; Neeb et al. 2017). These systems also use two distinct types of Argonaute family RNA-binding proteins to target regions of the genome for silencing or excision, using the AGOs or Piwis, respectively. AGOs are completely absent from ciliate genomes and Piwis have taken on the roles of these proteins. It remains unclear how Piwi-bound small RNAs “target” particular regions of the genome, but one can imagine the involvement of nascent transcripts within the developing macronucleus. Interestingly, plant AGO4 which binds siRNAs is most similar to Ptiwi10, known to bind iesRNAs in *Paramecium* during the second IES removal step of the “scanning model,” suggesting a possible similar mechanism (Furrer et al. 2017). Potentially, the most striking difference between these two systems is the fact that while both plants and ciliates silence particular regions of the genome using repressive histone modifications, plants merely form heterochromatin, while ciliates like *Tetrahymena* use repressive histone marks to excise and degrade large segments of the genome entirely, taking this process to the extreme. It remains to be elucidated whether ciliates and plants share additional commonalities in how they accomplish epigenetic inheritance and additional studies are needed to fill in the remaining gaps in our understanding of these models.

## Conclusion

Ciliates and plants represent unique and fascinating systems to study RNA-mediated transgenerational epigenetic inheritance. Both genomes must protect against the invasion of transposable elements and other foreign DNA, and this comes in the form of silencing expression, and in some cases excision, of entire DNA sequences to carefully defend the germline and subsequent generations. With the power of next-generation sequencing (NGS) of entire genomes and epigenomes, along with reverse genetic approaches, it will be possible to investigate the roles of epigenetic inheritance in other biological processes and contexts. Although transgenerational inheritance is clearly demonstrated and well described in ciliates and plants, further work is needed to investigate the implications in mammalian systems and how widespread this process is among other eukaryotes.
